# *Ralstonia pickettii* bacteremia in hemodialysis patients: a report of two cases

**DOI:** 10.5935/0103-507X.20160033

**Published:** 2016

**Authors:** Darwin Tejera, Gino Limongi, Mauricio Bertullo, Mario Cancela

**Affiliations:** 1 Asociación Española - Montevideo, Uruguay.

**Keywords:** *Ralstonia pickettii*/pathogenicity, Bacteremia/etiology, Intensive care units, Case reports

## Abstract

*Ralstonia pickettii* is a low-virulence gram-negative bacillus that may be associated with infections related to health care and may cause bacteremia. *Ralstonia pickettii* bacteremia is uncommon but is related to the contamination of medical products, mainly in immunodepressed patients. We present two cases of patients on chronic hemodialysis with *Ralstonia pickettii* bacteremia linked to contamination of the dialysis water. Similar cases have been published with links to intravenous fluid administration, medication ampules, and the use of extracorporeal oxygenation membranes, among other factors. The detection of *Ralstonia pickettii* bacteremia should provoke suspicion and a search for contaminated medical products, fluids, and/or medications.

## Abstract (pt)

*Ralstonia pickettii* es un bacilo gram negativo de baja virulencia que puede asociarse a infecciones relacionadas a los cuidados de la salud y provocar bacteriemias. La bacteriemia por *Ralstonia pickettii* es poco frecuente pero se relaciona con la contaminación de productos de uso médico principalmente en pacientes inmunodeprimidos. Presentamos dos casos en pacientes en hemodiálisis crónica vinculados a contaminación del agua de diálisis. Se han publicado casos similares vinculados a la administración de fluídos intravenosos, ampollas de medicación, asociado a membranas de circulación extracorpórea, entre otros. La detección de una bacteriemia por *Ralstonia pickettii*, debe sospechar e iniciar la búsqueda de productos de uso médico contaminados, fluídos y/o medicación.

## INTRODUCTION

*Ralstonia pickettii* is a gram-negative, aerobic, oxidase-positive, nonfermentative bacillus bacterium of the *Pseudomonas* group that is rarely associated with infections in humans. However, in recent years, *R. pickettii* has been identified as an emerging opportunistic pathogen. Although this is a low-virulence microorganism, it may be associated with infections related to health care, particularly as a cause of bacteremia. *R. pickettii* infections have been reported in a wide variety of patients, with symptoms ranging from asymptomatic clinical signs to severe sepsis with septic shock and death.^(1)^

## CASE REPORTS

### Clinical case 1

This patient was male, 65 years old, with chronic kidney disease, and on hemodialysis through a native arteriovenous fistula in the left arm. During the dialysis procedure, he developed general malaise and hypotension. The patient went to the emergency room; he was lucid, febrile, and polypneic, with sinus tachycardia, blood pressure of 60/40mmHg, peripheral coldness, and slow capillary refilling. The arteriovenous fistula did not exhibit fremitus or heartbeat. The findings of cardiovascular, respiratory, and abdominal examinations were normal.

Hemodynamic reanimation measures were initiated in the emergency room, two hemocultures were extracted, and admission to the intensive care unit (ICU) was requested.

In the ICU, the patient progressed to septic shock. Empirical treatment with vancomycin and meropenem began following a diagnosis of severe sepsis with no evident clinical focus. Paraclinical examinations showed the presence of severe metabolic acidosis with an arterial lactate level of 7mEq/L, anemia with a hemoglobin level of 8.1g/dL, and leukocytosis with a white blood cell count of 19,000/mL.

A transesophageal echocardiogram was requested, which ruled out endocarditis, and an echo-Doppler of the left arm showed thrombosis of the arteriovenous fistula.

On the third day of evolution, the hemoculture and dialysis water culture report was received, which showed *R. pickettii* growth. Vancomycin was suspended, and meropenem was maintained for 14 days. Good evolution followed, with ICU discharge on the sixth day.

### Clinical case 2

This patient was male, 45 years old, and had a personal history of smoking, hypertension, chronic kidney disease and kidney transplant with chronic rejection. The patient was on hemodialysis through a prosthetic arteriovenous fistula in the right arm. Twenty days prior to the episode, he developed signs of an upper respiratory infection and began receiving oral antibiotic treatment. The patient continued to experience febrile episodes and chills during the dialysis procedures; therefore, he was admitted to the general ward, two hemocultures were extracted, and treatment with ampicillin-sulbactam and ceftazidime was initiated. During clinical evolution, the patient presented with clinical bacteremia with hypotension, fever, and pulmonary edema; therefore, he was admitted to the ICU. Upon admission, the patient was lucid, febrile, hypotensive (blood pressure of 90/60mmHg), and well perfused, with sinus tachycardia, polypnea, and crepitant rales during pleuropulmonary auscultation. Fluid replacement, non-invasive mechanical ventilation, empirical antimicrobials, and emergency hemodialysis were initiated. The paraclinical assessment highlighted leukocytosis with a white blood cell count of 15,000/mL, anemia with a hemoglobin level of 8.9g/dL, and type 1 respiratory insufficiency with a PaO_2_/FiO_2_ index of 220.

A transthoracic echocardiogram was requested, which revealed the presence of a 2cm growth on the mitral valve. At 24 hours, a new transthoracic echocardiogram and a transesophageal echocardiogram were performed, which were normal and showed no growth. Hemocultures and a culture of the dialysis water grew *R. pickettii*. Antimicrobial treatment was adjusted to piperacillin-tazobactam, which was administered for 21 days. The patient had a good clinical evolution and was discharged from the ICU on the 12^th^ day.

## DISCUSSION

Gram-negative bacilli, primarily *Pseudomonas* species, are the microorganisms most frequently responsible for bacteremia in ICUs in Brazil.^(2)^

*Ralstonia pickettii* was first isolated in 1973 and included in the genus *Pseudomonas* spp. Subsequently, this microorganism was reclassified into the genus *Burkholderia*, and the genus *Ralstonia* was named in 1995. *R. pickettii* has been found in numerous sources of water, both domestic and in hospitals.^(3)^

*R. pickettii* has been identified as the causative agent of several serious infectious diseases, such as primary bacteremia, pneumonia, endocarditis, primary peritonitis, and infections associated with venous catheters, among others.^(3)^

The population at risk includes immunocompromised and hemato-oncology patients in the ICU with central venous catheters as well as newborns in the neonatal ICU.^(3)^

Many cases of infections with *Ralstonia* spp. are reported to be due to the use of contaminated solutions, blood products, chlorhexidine, saline solution, and sterile water, as well as the colonization of medical devices. Furthermore, hospital outbreaks due to the contamination of magnesium, ranitidine, and narcotics ampules have been reported.^(1)^

Contamination occurs during the manufacturing phase because the bacterium can pass through the 0.2 micron filters used for the sterilization of medicinal products.^(3)^*Ralstonia* spp. have low nutrient requirements and can survive in water and soil for long periods. Virulence occurs through the production of a biofilm that allows the microbe to remain in plastic pipes and medical devices^(1)^ and the ability to produce toxins. In health care centers in Brazil, *R. pickettii* has been identified in fluids for intravenous administration, resulting in the death of neonates and adult patients.^(4)^ In hospitals in North America, *R. pickettii* has been isolated from samples of tracheal secretions from patients on mechanical respiratory assistance; the source was contaminated 0.9% saline solution used for respiratory therapy.^(1)^

In the cases presented herein, the water used in hemodialysis was contaminated with *R. pickettii*, which was transmitted to the patients during the dialysis procedure. Similar cases linked to the contamination of dialysis water have been reported.^(5)^*Ralstonia* spp. grow in conventional media, although growth may be slow, and more than 72 hours of incubation may be required to visualize the colonies. This microorganism was identified in an automated BACTEC(tm) 9000 PLUS (Aerobic/FNMIC/ID-123) system; the positivization time of blood cultures was 2 to 3 days. The dialysis water was inoculated and cultured on blood agar in both cases. The time required to grow and identify the microorganisms was 3 to 4 days. Table 1 presents an antibiogram of the *R. pickettii* species isolated in both cases.

**Table 1:** Antibiogram of *Ralstonia pickettii* isolates in blood cultures and dialysis water in both clinical cases

**Antimicrobial**	**MIC (µg/ml)**	**Interpretation**
Amikacin	≤ 8	S
Aztreonam	> 16	R
Ceftazidime	> 16	R
Ciprofloxacin	≤ 0.5	S
Gentamicin	≤ 2	S
Imipenem	4	S
Levofloxacin	≤ 1	S
Meropenem	< 8	S
Piperacillin-tazobactam	4	S
Tobramycin	≤ 2	S
Trimethoprim-sulfamethoxazole	≤ 0.5/9.5	S

MIC - minimum inhibitory concentration; R - resistant; S - sensitive.

The presence of fever and bacteremia related to dialysis procedures should generate clinical suspicion of both arteriovenous fistula infection and dialysis water contamination. In the cases presented herein, the infection was cured without removal of the arteriovenous fistula. In other reported cases of *R. pickettii* bacteremia in patients with Port-a-Cath^®^, negativization of hemocultures was achieved only after removal of the devices.^(1)^

When the isolation of unusual microorganisms in hemocultures occurs, an active search should be initiated, with microbiological studies of all fluids, drugs used, and dialysis water in the case of hemodialysis patients. It should be noted that the contamination of hemoculture vials has been associated with the subsequent misidentification of bacteremia.^(1)^ In the ICU, such cases must be reported to the committee for infections and to local authorities to prevent and control outbreaks.

There are no standard recommendations for the treatment of infections with *Ralstonia* spp. with regard to drugs or duration. Differences are found in sensitivity to antibiotics, particularly carbapenems and aminoglycosides. The presence of two inducible beta-lactamases, blaOXA-60 and blaOXA-22, has been reported to be responsible for the high level of resistance to beta-lactams, and an aminoglycoside acetyltransferase accounts for the widespread resistance to aminoglycosides. However, the majority of the reported infections are treated with piperacillin-tazobactam, meropenem, ciprofloxacin, amikacin, or a combination of cephalosporins and aminoglycosides with good results.^(3)^ In the majority of the reports, antibiotic treatment was maintained for 14 days. In the second case presented herein, treatment was prolonged for 21 days based on the results of the first transthoracic echocardiogram, which indicated probable infectious endocarditis, although this was not confirmed by the second transthoracic echocardiogram or the transesophageal echocardiogram. Table 2 shows the main characteristics of *R. pickettii* bacteremia cases reported in adults in recent years.

**Table 2:** Main characteristics of adult cases of Ralstonia pickettii bacteremia reported in recent years

**Author/Year**	**Number** ** of cases**	**Primary site of infection**	**Comorbidities**	**Antibiotic treatment**	**Treatment duration (in days)**	**Mortality** ** (n of cases)**
Strateva et al., 2012(6)	1	Hemodialysis water	CKD and CHD	Ceftriaxone	N/D	N/D
Mikulska et al., 2009(7)	10	Not found	Hemato-oncology, chemotherapy, hematopoietic progenitor transplantation	Ceftriaxone Ceftazidime Ceftazidime + amikacin or carbapenem	N/D	1*
Pellegrino et al., 2008(4)	19	Intravenous fluids	Hemato-oncology Solid tumors	N/D	N/D	2*
Forgie et al., 2007(8)**	2	Extracorporeal oxygenation membrane (ECMO)	Congenital cardiopathy	Ciprofloxacin	14	0
Moreira et al., 2005(9)	14	Injection of contaminated water	N/D	Ciprofloxacin Gentamicin	N/D	0
Hsueh et al., 1998(10)	3	Catheter-related (Port-a-Cath^®^)	Hematologic neoplasia Polychemotherapy	Ciprofloxacin	7	0

CKD - chronic kidney disease; CHD - chronic hemodialysis; N/D - no data.

* Deaths not related to bacteremia.

** Pediatric patients.

## CONCLUSION

In conclusion, two cases of patients on chronic hemodialysis who developed severe sepsis from dialysis water contaminated with *Ralstonia pickettii* are presented. In both cases, the clinical evolution was good after the identification of the organism and specific antibiotic treatment for a minimum of 14 days. When bacteremia or another clinical isolate of *Ralstonia pickettii* is detected, medical product contamination should be suspected, and an epidemiological investigation should be initiated that includes microbiological examination of the administered fluids, medications, and hemodialysis water. Patients with endovascular devices and immunocompromised patients who present to the ICU with nonspecific infectious signs and severe sepsis should prompt special precautions and searches for infections by unusual organisms.
